# Prevalence of ultrasonography proved polycystic ovaries in North Indian women with type 2 diabetes mellitus

**DOI:** 10.1186/1477-7827-3-35

**Published:** 2005-08-11

**Authors:** Abdul H Zargar, Vipin K Gupta, Arshad I Wani, Shariq R Masoodi, Mir I Bashir, Bashir A Laway, Mohammad A Ganie, Mohammad Salahuddin

**Affiliations:** 1 Departments of Endocrinology and Immunology, Sheri-Kashmir Institute of Medical Sciences Srinagar, J&K, India

## Abstract

**Background:**

Polycystic ovaries (PCO) and their clinical expression (the polycystic ovary syndrome [PCOS]) as well as type 2 diabetes mellitus (T2DM) are common medical conditions linked through insulin resistance. We studied the prevalence of PCO and PCOS in women with diet and/or oral hypoglycemic treated T2DM and non-diabetic control women.

**Design:**

Prospective study.

**Methods:**

One hundred and five reproductive age group women with diet and /or oral hypoglycemic treated T2DM were the subjects of the study. Sixty age-matched non-diabetic women served as controls. Transabdominal ultrasonographic assessment of the ovaries was used to diagnose PCO. Clinical, biochemical and hormonal parameters were also noted.

**Results:**

Ultrasonographic prevalence of PCO was higher in women with diabetes than in non-diabetic subjects (61.0% vs. 36.7%, P < 0.003) whereas that of PCOS was 37.1% in diabetic subjects and 25% in non-diabetic controls (P > 0.1). Diabetic women with PCO had diabetes of significantly longer duration than those without PCO (4.19±2.0 versus 2.9±1.6 yrs; p < 0.05). Among both diabetic and non-diabetic women, those with PCO had significantly higher plasma LH, LH/FSH ratio, total testosterone and androstenedione levels.

**Conclusion:**

This study demonstrates a higher prevalence of PCO in women with T2DM as compared to non-diabetic subjects.

## Introduction

Polycystic ovary syndrome (PCOS) has, since its first description by Stein and Leventhal in 1935, become one of the commonest disorders in women, affecting 5 to 10% in the reproductive age-group [1-3]. This common medical condition has brought gynecologists, endocrinologists, cardiologists, pediatricians, and dermatologists together [4]. The disorder manifests as obesity, hirsutism, menstrual disturbances, acne vulgaris, male-pattern baldness, recurrent abortions, infertility, an-ovulation, and psychological and psychosexual morbidity [5]. It is an important cause of hirsutism and of infertility in our population [6,7]. With the advances and refinement in ultrasound technology, the presence of polycystic appearance of ovaries has increasingly been accepted as an essential part of the diagnosis of this syndrome and ultrasound suggestion of PCO has recently been introduced as one of the three diagnostic criteria of PCOS in Rotterdam consensus workshop 2003 [8]. Using the most conservative criteria for transabdominal studies, such as presence of ≥ 10 peripheral cysts per ovary, each 2–8 mm, and thickened stroma, some authors have shown that as many as 21–23% of normal female population have PCO on ultrasound [9-11]. Some women with otherwise confirmed PCOS may have normal sized ovaries on ultrasound and normal sized ovaries without increased folicllarity don't preclude the diagnosis in proper clinical settings [12-14]. The pathophysiology of PCOS, one of the commonest endocrinopathies, is far from clear. A plethora of data point to insulin resistance (IR) and hyperinsulinemia as the central factor, a suggestion supported by clinical benefits of insulin sensitizers [3,5,15,16].

T2DM, which accounts for 90–95% of all diabetes mellitus, is a common metabolic disorder, characterized by a combination of IR and insulin secretory defects, occurring in varying proportions [17]. Both T2DM and PCOS are now considered to be two metabolically similar but phenotypically different expressions of the same syndromic continuum with IR being the common and pivotal link, which may be genetically determined [15,18]. Women with PCOS are also at increased risk for cardiovascular disease (CVD), given the high prevalence of the metabolic syndrome among them [19]. Many studies have revealed an increased prevalence of various abnormalities of glucose tolerance in women with PCOS [3,20,21]. The scarcity of converse data on the prevalence of PCO among women with T2DM prompted this study on the prevalence of PCO in a cohort of premenopausal women with insulin naive T2DM.

## Subjects and Methods

The study was conducted in the department of Endocrinology of Sheri-Kashmir Institute of Medical Sciences Srinagar Kashmir (India), a tertiary care center. One hundred and sixty five premenopausal (≤ 45 years) women, from Kashmir region of India, were studied for sonographic suggestion of PCO in addition to recording of clinical, biochemical and hormonal manifestations of PCOS. Informed consent was obtained from all subjects and the institutional review board, that looks in to ethical aspects of human experimentation approved the study.

### Subjects

a. Patient group: This group comprised of 105 non-pregnant women with T2DM of at least one year duration treated with medical nutrition therapy and/or oral hypoglycemic drugs. Women with current or past history of insulin treatment, oral contraceptive use, and suspicion of nephropathy were excluded from the study.

b. Control group: Sixty non-diabetic, healthy pre-menopausal volunteers served as controls.

### Methods

Each patient and control received a detailed clinical examination and underwent a relevant laboratory evaluation.

a. Clinical assessment: The history focused on age, menstrual pattern, fertility status, duration and treatment of diabetes (in diabetic women), and other relevant drug history. Menstrual pattern was characterized as regular (cycles recurring every 21–35 days), oligommenorrhea (cycle length over 35 days and under six months), polymenorrhea (cycles occurring more frequently than every 21 days), menorrhagia (heavy menstruation requiring intervention) and amenorrhoea (absence of menstruation for six months or longer). Fertility status was classified as fertile (had a previous pregnancy with no subsequent infertility), infertile (primary or secondary infertility of at least 1 year duration) and unproven (pregnancy not attempted).

The physical examination, apart from a general review of the systems, focused on the assessment of androgen status (hirsutism, temporal recession of hair, acne, muscle bulk, clitoromegaly), evidence of IR (acanthosis nigricans), and anthropometry, (body mass index (BMI), waist circumference). Ferriman and Gallwey scoring system was used to assess hirsutism, [22]. and a score of 7 or more was taken as significant.

b. Laboratory evaluation. The baseline investigations included blood counts, chest x-ray, electrocardiogram, liver and kidney function tests, glucose and lipid (total cholesterol, HDL, and triglycerides) levels. A pool of three samples taken 15 minutes apart during early follicular phase was analyzed for LH, FSH, total testosterone and androstenedione levels. The blood sample for all investigations were taken in the fasting state in a single visit. The samples were taken in early follicular phase (day 3 – day 5) in women with regular cycles in all subjects and controls.

c. Ultrasonography. A specifically trained radiologist, using Siemens Sonoline Adara with 3.5 MHz convex electronic probe performed the transabdominal ultrasonography. Local socio-cultural constraints precluded a vaginal approach. The diagnosis of PCO was made, if 10 or more follicles, each 2–8 mm diameter were present in the ovarian periphery, and stroma was echo dense [23]. The ovarian volume was calculated by measuring the diameters in three dimensions and assuming an ellipsoid shape using the formula, volume = height × width × depth × π/6.

d. Hormonal assays. All hormone estimations were done with radioimmunoassay. Total testosterone and androstenedione were measured using commercial kits from DPC, Los Angeles CA. Serum LH and FSH was assayed by immunoradiometric assays using kits from Medicorp Inc., Montreal, Canada. Sensitivity, specificity, and inter- and intra-assay coefficients of variation were within the prescribed limit as provided by the manufacturer.

### Statistical analysis

SPSS 10.0 package was used for analysis of data. Correlations were done by Pearson's test. Categorical variables were compared by using Chi-square test, and for continuous variables, '*t*' test was used for comparing two groups and one way ANOVA when more than two groups were involved. Two tailed significance was calculated and a *P *value of <0.05 was taken as significant.

## Results

The baseline characteristics of diabetic and control women (Table 1) were comparable except for significantly higher systolic and diastolic blood pressures in the former. Table 2 compares the various metabolic and hormonal parameters between diabetic and non-diabetic women. While the mean fasting blood glucose and LDL levels were significantly higher in diabetic women, the mean levels of cholesterol, HDL, triglycerides and VLDL were similar in diabetic and control groups. The diabetic women had a significantly higher mean ovarian volume than controls (6.15 ± 1.4 ml vs. 5.66 ± 1.4 ml, P < 0.04). On ultrasonography, PCO was found in 64 (60.9%) and 22 (36.7%) of diabetic and control women respectively, giving a significantly higher prevalence in the diabetic women (P < 0.05). In women with PCO, those with and without T2DM had comparable mean ovarian volumes (5.80 ± 1.18 vs. 5.98 ± 1.28 ml; P > 0.5). Among women with T2DM, those with PCO had a significantly higher mean ovarian volume than those without PCO (5.80 ± 1.18 vs. 4.13 ± 1.07 ml; P < 0.001).

**Table 1:** Clinical profile of study subjects

**Clinical parameter**	**Patients (n = 105)**	**Controls (n = 60)**	**p**
Age (yrs)	36.19 ± 4.37*	34.98 ± 4.60	0.096
SBP (mmHg)	138.95 ± 12.39	132.75 ± 10.17	0.01^†^
DBP (mmHg)	84.84 ± 7.72	81.13 ± 8.57	0.05^†^
BMI (kg/m2)	25.42 ± 3.2	25.72 ± 3.2	0.57
W/H ratio	0.94 ± 0.7	0.93 ± 0.6	0.92

* Mean ± SD; †Significant

**Table 2:** Metabolic and hormonal profile of study subjects

**Parameter**	**Patients (n = 105)**	**Controls (n = 60)**	**P**
Fasting glucose (mg/dl)	120.61 ± 34.79*	88.07 ± 14.18	0.001^†^
Total cholesterol (mg/dl)	193.51 ± 47.88	183.63 ± 39.72	0.177
Triglycerides (mg/dl)	193.69 ± 68.80	185.68 ± 70.89	0.478
LDL cholesterol (mg/dl)	128.96 ± 61.9	102.78 ± 40.1	0.004^†^
HDL cholesterol (mg/dl)	42.52 ± 7.56	43.99 ± 8.92	0.262
VLDL cholesterol (mg/dl)	38.7 ± 13.7	37.1 ± 14.1	0.478
LH (IU/L)	5.91 ± 5.72	4.67 ± 6.44	0.20
LH/FSH	1.01 ± 0.73	0.84 ± 0.66	0.132
Testosterone (ng/ml)	0.29 ± 0.22	0.27 ± 0.14	0.335
Androstendione (ng/ml)	1.87 ± 1.70	1.54 ± 0.64	0.157

* Mean ± SD; ^†^Significant

Table 3 shows clinical features in subjects with and without PCO on ultrasonography in both groups. Overall, PCO-syndrome (PCOS) was seen in 39 (37.1%) subjects with T2DM and 15 (25.0%) controls. However, this difference was not statistically significant (P > 0.1). The mean duration of diabetes and mean serum LH, testosterone, androstenedione, and LH/FSH ratio were significantly higher in diabetic women with than in those without PCO (Tables 3 &4). While non diabetic controls with and without PCO had comparable age, age at menarche, diastolic blood pressure, BMI and waist hip ratios, the former had a significantly higher mean systolic blood pressure (Table 3). As in the diabetic women, non-diabetic controls with PCO had higher LH, LH/FSH ratio, testosterone and androstenedione levels than those without PCO; among both diabetic and non diabetic women, the blood glucose, and lipid levels were comparable in those with and without PCO (Table 4). The clinical and biochemical parameters of diabetic and non-diabetic women with PCO were comparable (Tables 3 &4). Of 105 diabetic subjects, 55 were overweight-obese (BMI ≥ 25) whereas of 60 controls, 30 were overweight-obese. Table 5 shows clinical features and frequency of PCO and PCOS in these obese and non-obese subjects.

**Table 3:** Clinical profile of diabetic patients and controls with and without PCO

**Clinical Parameter**	**Diabetic subjects**		**Controls**		**P value (ANOVA)**
		
	**PCO (n = 64)**	**Non-PCO (n = 41)**	**PCO (n = 22)**	**Non-PCO (n = 38)**	
Age (years), Mean ± SD	36.8 ± 4.28*	35.2 ± 4.4	34.2 ± 4.5	35.5 ± 4.6	0.000
Duration of DM (y), Mean ± SD	4.19 ± 2.0	2.9 ± 1.6 ^a^	--	--	0.000
Age of menarche (y), Mean ± SD	13.5 ± 0.8	13.50 ± 0.6	13.1 ± 0.8	13.1 ± 0.7	0.015
Menstrual irregularity,	24 (37.5%)	4 (9.8%)	0	0	0.000
Oligomenorrhea	12 (18.8%)	1 (2.4%)			
Polymenorrhea	1 (1.6%)	1 (2.4%)			
Mennorrhagia	11 (17.2%)	2 (4.9%)			
Infertility	4 (6.2%)	0	0	0	0.368
Hirsuitism	9 (14.1%)	0	4 (18.2%)	2 (5.3%)	0.030
Acne	3 (4.7%)	0	1 (4.5%)	1 (2.6%)	0.564
Acanthosis	2 (3.1%)	0	0	0	0.368
History of hypertension	40 (78%)	30 (73%)	0	0	0.000
SBP (mmHg), Mean ± SD	140 ± 12.7	139 ± 12.3	138 ± 9.5	130 ± 9.3 ^b^	0.000
DBP (mmHg), Mean ± SD	85.8 ± 7.8	83.3 ± 7.3	82.1 ± 10.2	80.58 ± 7.5	0.012
BMI (kg/m2), Mean ± SD	25.8 ± 3.1	24.7 ± 3.3	26.36 ± 2.8	25.34 ± 3.3	0.180
Waist to hip ratio, Mean ± SD	0.94 ± 0.6	0.92 ± 0.7	0.94 ± 0.6	0.92 ± 0.6	0.426
Ovarian volume (ml), Mean ± SD	5.80 ± 1.18	4.13 ± 1.07	5.98 ± 1.28	3.89 ± 0.88	0.000
PCOS^†^	39 (61%)	0	15 (68%)	0	0.000

* on ultrasonography

^a^: P < 0.05, in the subject group, ^b^: P < 0.05, in control group

^† ^PCOS by Rotterdam ASRM/ESHRE consensus criteria

**Table 4:** Showing comparison of metabolic and hormonal profile in diabetic subjects and non-diabetic controls on the basis of presence or absence of ultrasonic suggestion of polycystic ovaries (PCO).

**Clinical parameter**	**Diabetic subjects**		**Controls**		***P ***(ANOVA)
		
	**PCO **(n = 64)	**Non-PCO **(n = 41)	**PCO **(n = 22)	**Non-PCO **(n = 38)	
Fasting glucose (mg/dl)	123.8 ± 36.28	115.55 ± 32.0	92.14 ± 12.7	85.71 ± 14.6	0.000
Total cholesterol (mg/dl)	189.4 ± 40.9	199.8 ± 57.0	183.27 ± 37.6	183.87 ± 41.3	0.375
Triglycerides (mg/dl)	194.4 ± 69.9	192.4 ± 67.8	182.27 ± 52.1	187.65 ± 80.2	0.895
HDL cholesterol (mg/dl)	42.0 ± 6.6	43.3 ± 8.8	44.00 ± 11.0	43.99 ± 7.6	0.593
LDL cholesterol (mg/dl)	127.5 ± 55.2	131.1 ± 71.9	97.74 ± 40.7	105.76 ± 40.0	0.033
VLDL cholesterol (mg/dl)	38.8 ± 13.9	38.4 ± 13.5	36.45+10.4	37.53+16.0	0.895
LH (IU/L)	7.51+6.65	3.42 +2.21^a^	7.93 ± 9.52	2.77 ± 2.17^b^	0.000
FSH (IU/L)	5.82+2.80	7.23+4.86	5.50 ± 2.3	5.11 ± 2.9	0.039
LH/FSH ratio	1.31 ± 0.78	0.55 ± 0.26^a^	1.3 ± 0.85	0.5 ± 0.26^b^	0.000
Testosterone (ng/ml)	37.4 ± 23.0	16.9 ± 7.3^a^	39.6 ± 13.3	18.8 ± 7.5^b^	0.000
Androstendione (ng/ml)	2.40 ± 1.9	1.04 ± 0.36^a^	2.10 ± 0.6	1.21 ± 0.40^b^	0.000

* Mean ± SD

^a^: P < 0.05, in the subject group, ^b^: P < 0.05, in control group

**Table 5:** Clinical features and frequency of PCO/PCOS in obese and non-obese subjects

**Characteristic**	**Diabetic cases**		**Controls**		**P value**
		
	**Obese **(n = 55)	**Non-obese **(n = 50)	**Obese **(n = 30)	**Non-obese **(n = 30)	
**Menstrual irregularity**					
Oligomenorrhea	9 (16.4%)	4 (8.0%)	0	0	0.014
Polymenorrhea	2 (3.6%)	0	0	0	0.256
Mennorrhagia	9 (16.4%)	4 (8.0%)	0	0	0.014
**Hirsutism**	7 (12.7%)	2 (4.0%)	2 (6.7%)	4 (13.3%)	0.346
**Acne**	2 (3.6%)	1 (2.0%)	1 (3.3%)	1 (3.3%)	0.966
**Acanthosis nigricans**	2 (3.6%)	0	0	0	0.256
**PCO**	34 (61.8%)	30 (60.0%)	14 (46.7%)	8 (26.7%)	0.009
**PCOS**	22 (40.0%)	17 (34.0%)	10 (33.3%)	8 (16.7%)	0.181

## Discussion

PCOS and T2DM are common medical conditions and both are associated with IR and compensatory hyperinsulinemia [24]. The clinical and biochemical features of PCOS are heterogeneous and there is much debate whether it represents a single or several disorders [2]. In recent years it has become apparent that PCOS not only is the most frequent cause of anovulation and hirsutism, but also is associated with characteristic metabolic disturbances that may have implications for long-term health [2,4]. Recently IR has been clearly documented by many investigators in PCOS, although the cause of IR is unknown and its relationship with PCOS as an etiological agent or an epiphenomenon remains unsettled [3,15,25,26]. T2DM is a common disorder characterized by IR and compensatory hyperinsulinemia [17]. An increased incidence of glucose intolerance and DM has been reported in women with a history of PCOS compared to controls in long-term studies [3,20,21]. If IR and hyperinsulinemia have an important role in determining ovarian morphology, PCO and their clinical expression can be expected to be more common among women with T2DM. We compared the prevalence of PCO on ultrasound in a defined clinic population of women with T2DM and in a group of age matched non-diabetic control women.

In our study 60.95% of the 105 premenopausal women with T2DM were documented to have PCO compared to 36.66% of the 60 non-diabetic controls. This prevalence in our study is much higher than the reported prevalence of 21–23% among normal female population [9-11]. In previous studies we observed PCOS to be second common cause of both hirsutism and infertility in our population [6,7]. Rodin et al reported an overall prevalence of 52% of PCO in women of Indian subcontinent origin living in England [27]. Conn et al studied 38 premenopausal women using the same ultrasonographic criteria and reported prevalence 82% among women with T2DM [24]. Not much data are available on the prevalence of PCO among diabetic women.

In our study diabetic females with PCO had significantly higher mean LH, total testosterone and androstenedione than diabetic females without PCO. Conn and colleagues found comparable mean levels of LH and testosterone in diabetic women with and without PCO and significantly higher levels of androstenedione in the former [24]. Another study, including women with and without diabetes, found significantly higher mean testosterone levels in women with PCO than in those without [27]. No statistically significant differences were found between mean systolic and diastolic blood pressures, BMI, waist hip ratios, HDL-cholesterol and triglyceride levels in diabetic women with and without PCO like observations made earlier by Conn et al [24]. We did not find any difference in the prevalence of PCO among obese (BMI ≥ 25 kg/m^2^) and non-obese (BMI <25 kg/m^2^) women with T2DM. No study has compared the prevalence of PCO among obese and non-obese type 2 diabetic women. As in the patient group, non-diabetic controls with PCO had significantly higher mean LH, testosterone and androstenedione levels and mean ovarian volume than those without PCO; all clinical and metabolic parameters were comparable between these two subgroups of non-diabetic controls except mean systolic blood pressure which was significantly more among controls with than without PCO (138.1 vs. 129.6 mmHg).

We found a significantly higher mean LDL levels in: a) diabetic women than non-diabetic women (128 mg/dl vs. 102 mg/dl), b) women with PCO and T2DM than women with PCO and without T2DM (127 mg/dl vs. 97 mg/dl) and c) obese women with PCO and T2DM than obese women with PCO but without T2DM (139 mg/dl vs. 87 mg/dl). This likely reflects the effect of diabetes on plasma lipids. An earlier study by Rodin found no difference in respect to LDL, HDL, triglycerides or total cholesterol in women of Indian sub continental ancestry with and without PCO [27]. Comparing women with PCO from the diabetic and control groups, a significant difference was found in the mean age of women from these two groups (36.83 vs. 34.18 years; P < 0.02). The mean LDL level in women with T2DM and PCO was significantly higher than in controls with PCO (127 vs. 97 mg/dl; *P *< 0.03); other parameters including diastolic blood pressure, BMI, waist hip ratio, cholesterol, triglycerides, HDL, VLDL, LH, testosterone and androstenedione and ovarian volume were comparable between women with PCO and with or without T2DM.

The significantly longer duration of diabetes among diabetic patients with PCO compared to those without PCO in our study probably reflects longer duration of insulin resistance/hyperinsulinemia in the former. IR is considered central to the pathogenesis of PCOS and south Asian women with PCOS have been reported to be more insulin resistant, seek treatment at a younger age and have more severe symptoms than Caucasians [28]. Hyperinsulinemia augments androgen production in PCOS directly by augmenting LH activity through stimulation of ovarian receptors of insulin and insulin-like growth factors or indirectly, by enhancing the amplitude of serum LH pulses [5]. Furthermore, hyperinsulinemia decreases SHBG levels increasing the amount of free testosterone available to act on target organs [24]. Legro and colleagues reported that 50–70% of women with PCOS have IR which probably contributes to hyperandrogenism underlying the signs and symptoms of PCOS [29]. While IR is a common feature of both PCOS and T2DM, persistent reproductive dysfunction appears to be limited to the former raising the possibility that IR in the ovary itself may confer this susceptibility. The exact role and mechanism(s) thereof IR and hyperinsulinemia are far from fully understood. While Conn et al,24 found fasting insulin levels in diabetic women with and without PCO to be comparable, Rodin et al [27]. observed in a more genetically homogenous group of Asian women, that those with both PCO and T2DM were more insulin resistant than those with diabetes alone.

In conclusion our study demonstrates a higher prevalence of PCO and its clinical expressions in women with T2DM compared to non-diabetics. The precise role of hyperinsulinemia in development and expression of PCO remains largely unresolved and needs elucidation.
